# CLINICAL, DIAGNOSTIC AND THERAPEUTIC CHARACTERIZATION OF PATIENTS WITH PANCREATIC COLLECTIONS DUE TO ACUTE PANCREATITIS IN A REFERRAL HOSPITAL

**DOI:** 10.1590/S0004-2803.24612024-105

**Published:** 2025-07-21

**Authors:** Rómulo Darío VARGAS, Ana María LEGUIZAMO-NARANJO, Oscar Mauricio MUÑOZ-VELANDIA, Rafael Gregorio PEÑA-AMAYA

**Affiliations:** 1 Gastroenterology and Digestive Endoscopy Unit, Hospital Universitario San Ignacio, Bogotá, Colombia.; 2 Department of Internal Medicine, Pontificia Universidad Javeriana, Bogotá, Colombia.; 3 Department of Internal Medicine, Hospital Universitario San Ignacio, Bogotá, Colombia.

**Keywords:** Acute pancreatitis, walled -off necrosis, acute necrotic collection, acute fluid collection, Pancreatite aguda, necrose isolada, coleção necrótica aguda, coleção aguda de fluidos

## Abstract

**Background:**

Pancreatic collections are a common complication of acute pancreatitis. In Latin America, information on the types of pancreatic collections and their management is limited and may vary between regions depending on the availability of highly specialised and minimally invasive treatment resources.

**Methods:**

Retrospective cohort of patients with acute pancreatic collections secondary to acute pancreatitis treated at the Hospital Universitario San Ignacio, Bogotá (Colombia) between 2012-2023. Clinical characteristics, laboratory profiles and treatment received were described, comparing those who had a fatal outcome with those who did not.

**Results:**

Of 689 patients with acute pancreatitis, 113 presented with pancreatic collection (55.1% women, mean age 55 years). Of these, 47.8% presented with acute necrotic collection, 36.3% with acute fluid collection, 9.7% with walled-off necrosis and 6.2% with pancreatic pseudocyst. C-reactive protein, BUN, creatinine levels (at admission and at 48 hours), PaO2/FiO2 (at admission and at 48 hours) and antibiotic use were significantly associated with mortality (*P*<0.05). The majority of acute necrotic collections, walled-off necrosis and pseudocysts received interventional management, with minimally invasive and combined management being more common than surgical management. Antibiotic management was used in 48.6% of collections, although microbiological isolation was performed in only 24.7% of cases.

**Conclusion:**

Acute collections are a common and heterogeneous complication of pancreatitis, requiring intervention more often in complicated collections. Certain laboratory parameters seem to be more associated with mortality.

## HIGHLIGHTS

• Pancreatic collections are a common complication of acute pancreatitis.

• Their management depends on the availability of specialized and minimally invasive resources.

• A study conducted on 689 patients with acute pancreatitis showed that 113 developed pancreatic collections.

• Acute pancreatic collections are frequent and heterogeneous complications of pancreatitis.

• Some altered laboratory parameters (creatinine, urea, C-reactive protein, and FiO_2_) seem to be more associated with mortality.

## RESUMO

**Contexto:**

Coleções pancreáticas são uma complicação comum da pancreatite aguda. Na América Latina, as informações sobre os tipos de coleções pancreáticas e seu manejo são limitadas e podem variar entre regiões, dependendo da disponibilidade de recursos especializados e minimamente invasivos.

**Métodos:**

Estudo de coorte retrospectivo com pacientes apresentando coleções pancreáticas secundárias à pancreatite aguda, atendidos no Hospital Universitário San Ignacio, Bogotá (Colômbia), entre 2012 e 2023. Foram descritas características clínicas, perfis laboratoriais e tratamentos recebidos, comparando pacientes com e sem desfecho fatal.

**Resultados:**

Dos 689 pacientes com pancreatite aguda, 113 desenvolveram coleções pancreáticas (55,1% mulheres, idade média de 55 anos). Entre eles, 47,8% apresentaram coleção necrótica aguda, 36,3% coleção líquida aguda, 9,7% necrose encapsulada e 6,2% pseudocisto pancreático. Níveis de proteína C reativa, ureia, creatinina (na admissão e após 48 horas), PaO2/FiO2 (na admissão e após 48 horas) e uso de antibióticos foram significativamente associados à mortalidade (*P*<0,05). A maioria das coleções necróticas agudas, necrose encapsulada e pseudocistos recebeu tratamento intervencionista, sendo os métodos minimamente invasivos e combinados mais comuns do que a abordagem cirúrgica. O uso de antibióticos ocorreu em 48,6% dos casos, embora o isolamento microbiológico tenha sido realizado em apenas 24,7% dos pacientes.

**Conclusão:**

As coleções pancreáticas agudas são complicações frequentes e heterogêneas da pancreatite, exigindo intervenção principalmente em casos mais complexos. Alguns parâmetros laboratoriais parecem estar mais associados à mortalidade.

## INTRODUCTION

Acute pancreatitis is one of the leading causes of emergency department visits and gastroenterology hospitalizations worldwide, associated with a high socioeconomic burden. In the United States it accounts for approximately 275,000 admissions per year at a cost of $2.5 billion annually^1^. In Latin America, hospitalization costs per patient have been reported to be as high as US$ 15,000 per year^2^ in Colombia, while another study reported annual costs of US$ 2.54 billion^3^ in Chile, suggesting costs similar to those reported in the United States. Pancreatic collections are a common complication of acute pancreatitis, 5-15% of pancreatitis episodes presenting with pseudocyst development as a complication, 15% with pancreatic necrosis, and approximately 33% (with a range of 16-47%) with infected necrosis^4^.

The local complication with the highest mortality is necrotic collection, estimated at 10-36% of cases, a percentage that has persisted despite advances in critical care^5^. The main determinants of morbidity and mortality are the presence of organ failure and necrosis. Organ failure is related to the degree of necrosis and the presence of local or systemic infection^6^. Secondary infection of necrotic tissue occurs in 30-70% of patients with acute necrotic collection and is associated with worse outcomes^6^. Recent systematic reviews report a mortality rate of 35.2% in patients with infected necrosis and organ failure, while non-infected necrosis associated with organ failure had a mortality rate of 19.8%. Patients with infected necrosis without organ failure have a mortality rate of 1.4%^7^.

The management of pancreatic complications has changed over the past two decades. In the past, surgical management was the most common approach, whereas currently the literature advocates a bottom-up approach starting with minimally invasive therapy, with surgical management reserved for cases in which minimally invasive management was unsuccessful or ineffective^4^ ^,^ ^8^. The American College of Gastroenterology (ACG) guidelines recommend minimally invasive necrosectomy over open surgical necrosectomy for symptomatic patients with infected necrosis^4^. The British Society of Gastroenterology^9^ guidelines recommends minimally invasive drainage for the management of acute infected necrotic collection and walled-off necrosis, followed by surgical intervention only when necessary and indicated. Minimally invasive management includes percutaneous puncture by interventional radiology and endoscopic management guided by endoscopic ultrasound^10^. However, in Latin America and Colombia there are no comprehensive data on the management of acute pancreatic collections and adherence to the recommendations of international guidelines. A study conducted in Mexico and published in 2021, reported that 87.8% of physicians and residents attached to the gastroenterology service considered that infected necrosis has a surgical indication^11^, suggesting a lack of knowledge or adherence to current recommendations.

In our setting, there may be differences in recommended management due to the limited availability of resources for minimally invasive management, which is highly specialized. The present study aims to characterize and describe the demographic, clinical and diagnostic variables, type of pancreatic collections, treatment used and mortality in patients older than 18 years with acute pancreatic collections secondary to acute pancreatitis in a referral hospital in Colombia.

## METHODS

Retrospective observational cohort study that included all patients who developed acute pancreatic collections (acute fluid collection, acute necrotic collection, pancreatic pseudocyst, and walled-off necrosis) during hospitalization at Hospital Universitario San Ignacio (HUSI), Bogota, Colombia, between January 2012 and September 2023. Patients over 18 years of age with a diagnosis of pancreatic collections secondary to acute pancreatitis who had two of the following three characteristics: characteristic abdominal pain, biochemical evidence of pancreatitis (elevated amylase or lipase >3 times the upper limit of normal), or radiologic evidence of pancreatitis on sectoral imaging were included. Patients with acute myocardial infarction in the previous three months, decompensated heart failure, decompensated liver disease, chronic kidney disease, or radiologic evidence of chronic pancreatitis were excluded from the study because they could bias the mortality estimate. Patients were also excluded if they were referred to another institution for treatment of acute pancreatitis. The study was approved by the Ethics Committee of the HUSI and the Pontificia Universidad Javeriana (032-2020).

Patients were identified from the database of patients hospitalized by the Internal Medicine and Gastroenterology service with a diagnosis of acute pancreatitis, corroborating the non-repetition of the patients included. After reviewing the inclusion and exclusion criteria, information was collected from the electronic medical record using a standardized format, including demographic variables, comorbidities, need for intensive care unit (ICU) stay, presence of organ dysfunction, duration of organ dysfunction, mortality, etiology of mortality, type of acute pancreatic collection, antibiotic use, management of acute pancreatic collections, microbiological isolation, management with crystalloids in the first 24 hours, and the results of the diagnostic test performed.

Pancreatic collections were defined according to the revised Atlanta criteria: acute fluid collection, acute necrotic collection, pancreatic pseudocyst, and walled-off necrosis defined by computed tomography or magnetic resonance imaging^12^. Organ dysfunction was defined by the modified Marshall score^13^. Minimally invasive management of pancreatic collections referred to those performed percutaneously by interventional radiology or endoscopically.

For statistical analysis, demographic characteristics were described using measures of central tendency and measures of dispersion, mean and standard deviation for those characteristics with a normal distribution, or median and interquartile range (IQR) for those continuous variables with a non-normal distribution. The Shapiro-Wilk test was used to evaluate the assumption of normality. Categorical variables are presented as absolute numbers and percentages. The t-test was used to compare continuous variables with normal distribution, Mann-Whitney u test was used to compare continuous variables without normal distribution and χ² test was used to compare groups according to mortality. Statistical analysis was performed using STATA 16® statistical package (StataCorp, College Station, TX, USA).

## RESULTS

We identified 689 patients with acute pancreatitis criteria, of which 113 patients had pancreatic collections and were included in the analysis. The primary etiology of pancreatitis was biliary (72.6%), followed by idiopathic (4.4%), alcoholic (2.7%), hypertriglyceridemia (2.7%), and medication-related (2.7%). In 10.6% of cases, the etiology remained undetermined. The sociodemographic characteristics and outcomes of the patients are shown in Table 1. Of the total number of patients, 55.1% were female, median age was 55 years (IQR: 37-65). 36.3% (n=41) of patients developed acute fluid collection, 47.8% (n=54) acute necrotic collection, 6.2% (n=7) pseudocyst and 9.7% (n=11) walled-off necrosis. The most common comorbidity was systemic arterial hypertension (25.66%), followed by dyslipidemia (15.04%) and alcoholism (10.62%). 46.9% of the patients required ICU stay and 12.3% died (n=14). Of these, three were in the acute fluid collection group and 11 had acute necrotic collection. Four patients died due to infected collection, 4 due to other causes and 6 due to both causes. Table 2 shows the management of pancreatic collections. 48.67% of the collections (n=55) received antibiotic management. The most frequent management was surveillance without intervention (56.7%), followed by minimally invasive drainage (22.1%), combined drainage (13.3%) and surgical drainage (7.9%). There were differences in management according to type of collection, with walled-off necrosis requiring interventional management, either minimally invasive or combined, in 100% of cases. Likewise, acute necrotic collections and pancreatic pseudocysts were managed with active drainage in more than half of the cases. The minority of collections had microbiological isolation (24.78%).

**TABLE 1:** Sociodemographic characteristics and outcomes of patients with pancreatic collections secondary to acute pancreatitis according to type of complication.

Variable	Total	Acute fluid collection	Acute necrotic collection	Pseudocyst	Walled necrosis
	(n=113)	(n=41)	(n=54)	(n=7)	(n=11)
Age in years, median (IQR)	55 (37-65)	50 (34-62)	57-5 (42-69)	42 (22-72)	54 (49-62)
Female sex, n (%)	60 (55.1)	23 (56.1)	31 (57.4)	3 (42.8)	3 (27.3)
**Comorbidity, n (%)**					
Systemic arterial hypertension	29(25.7)	10 (24.4)	16 (29.63)	2 (28.6)	1 (9.1)
Coronary heart disease	3 (2.6)	1 (2.4)	2 (3.7)	0 (0)	0 (0)
Stroke	2 (1.7)	2 (4.8)	0 (0)	0 (0)	0 (0)
Hypothyroidism	7 (6.2)	1 (2.4)	6 (11.1)	0 (0)	0 (0)
Dyslipidemia	17(15.0)	2 (4.8)	14 (25.9)	1 (14.3)	0 (0)
Acid peptic disease	7 (6.2)	4 (9.7)	3 (5.56)	0 (0)	0 (0)
Neoplasia	5 (4.4)	1 (2.4)	4 (4.7)	0 (0)	0 (0)
Type 2 diabetes	10(8.8)	2 (4.9)	6 (11.1)	0 (0)	2 (18.2)
Autoimmune disease	2 (1.7)	1 (2.4)	1 (1.8)	0 (0)	0 (0)
Alcoholism	12(10.6)	5 (12.2)	5 (14.3)	1 (14.3)	1 (9.1)
ICU requirement, n (%)	53 (46.9)	7 (17.1)	39 (72.2)	1 (14.9)	6 (54.5)
**Organ dysfunction, median (IQR)**					
PaO2/FiO2 on admission		300 (266-328)	286.5(257-332)	315 (242-323)	314 (271-328)
PaO2/FiO2 at 48 hours		266 (242-323)	250 (200-301)	280 (233-319)	257 (216-330)
Creatinine inlet		0.79(0.7-0.99)	0.88 (0.65-1.06)	0.74 (0.71-1.23)	0.84 (0.68-0.97)
Creatinine at 48 hours		0.74(0.61-0.93)	0.88 (0.63-1.14)	0.73 (0.56-0.75)	0.82 (0.7-1.65)
**Circulatory dysfunction, n (%)**					
SBP >90 mmHg	94(82.3)	34 (82.9)	45 (83.3)	5 (71.4)	9 (81.8)
SBP < 90 mmHg	15(13.3)	5 (12.2)	7 (12.9)	2 (28.5)	1 (9.1)
SBP < 90 mmHg without response to crystalloids	5(4.4)	2 (4.8)	2 (3.7)	0 (0)	1 (9.1)
Death, n (%)	14(12.3)	3 (7.31)	11 (20.37)	0 (0)	0 (0)
**Etiology of death, n (%)**					
Infected collection	4 (3.5)	0 (0)	4 (7.4)	0 (0)	0 (0)
Other causes	4 (3.5)	3 (7.3)	1 (1.8)	0 (0)	0 (0)
Both	6 (5.3)	0 (0)	6 (11.1)	0 (0)	0 (0)

ICU: intensive care unit; SBP: systolic blood pressure; IQR: interqurtilic range.

**TABLE 2:** Management of acute pancreatic collections secondary to acute pancreatitis.

Variable	Total	Acute fluid collection	Acute necrotic collection	Pseudocyst	Walled necrosis
	(n=113)	(n=41)	(n=54)	(n=7)	(n=11)
Antibiotic use, n (%)	55 (48.7)	9 (21.9)	34 (62.9)	2 (28.6)	10 (90.9)
**Collection management, n (%)**					
Surveillance without intervention	64 (56.7)	36 (87.8)	25 (46.3)	3 (42.8)	0 (0)
Minimally invasive drainage	25 (22.1)	5 (12.2)	11 (20.4)	3 (42.8)	6 (54.5)
Surgical drainage	9 (7.9)	0 (0)	8 (14.81)	1 (14.3)	0 (0)
Combined drainage	15 (13.3)	0 (0)	10 (18.5)	0 (0)	5 (45.4)
Microbiological isolation, n (%)	28 (24.8)	2 (4.88)	17 (31.5)	1 (14.3)	3 (27.3)
**Volume of crystalloids administered in the first 24 hours, n (%)**					
0-1000 mL	15 (13.2)	5 (12.2)	6 (11.1)	0 (0)	4 (36.4)
1001-3000 mL	67 (59.3)	26 (63.4)	31 (57.4)	5 (71.4)	5 (45.4)
>3000 mL	31 (27.4)	10 (24.4)	17 (31.5)	2 (28.6)	2 (18.2)

Table 3 shows the results of the diagnostic test performed according to the type of pancreatic collection. Within these, CRP levels were higher in patients with acute necrotic collection (median 23.2 mg/dL, IQR 14.8-26.2), as were levels of leukocytes, absolute neutrophils, creatinine (median 0.88 mg/dL, IQR 0.65-1.06), and BUN (mean 15.9 mg/dL, IQR 11.8-21.2). Strikingly, patients with acute fluid collections had higher levels of lipase (median 1145 mg/dL, IQR 676-7214), ALT (median 128 mg/dL, IQR 45-496.5), AST (median 118 mg/dL, IQR 47-367.5) and total bilirubin (median 1.75 mg/dL, IQR 0.88-3.655) compared to the other collections.

**TABLE 3:** Results of the diagnostic test performed according to type of pancreatic collection.

Variable	Acute fluid collection	Acute necrotic collection	Pseudocyst	Walled necrosis
	(n=41)	(n=54)	(n=7)	(n=11)
CRP mg/dL, median (IQR)	11.45 (6.9-21.9)	23.2 (14.8-26.2)	9.275 (8.5-19.6)	17.6 (8.6-31.7)
Leukocytes (mm3), median (IQR)	11900 (9180-16070)	14920 (11400-20600)	14720 (10800-15600)	15400 (10400-17835)
Neutrophils (mm3), median (IQR)	10860 (6970-14190)	12835 (9000-18200)	11600 (9100-13500)	13500 (7800-14400)
Hemoglobin (g/dL)	14.6 (12.9-15.9)	15.8 (13.8-17.4)	14.39 (10.9-15.3)	12.6 (10.6-16.9)
Platelets (mm3), median (IQR)	317300	252950	326000	268500
	(234000-392100)	(205500-335400)	(252000-384400)	(205100-563000)
Amylase mg/dL, median (IQR)	1565 (588-2744)	1618 (552-3328)	841 (415-1176)	71 (40-2010)
Lipase mg/dL, median (IQR)	1145 (676-7214)	713 (248-4703)	613 (319.3-640)	869 (117-1051)
ALT mg/dL, median (IQR)	128 (45-496.5)	100 (32-263)	35 (23-115)	36 (14-68)
AST mg/dL, median (IQR)	118 (47-367.5)	77.5 (30-331)	34 (19-159)	33 (15-106)
Bilirubin levels in mg/dL, median (IQR)				
Total bilirubin	1.75 (0.88-3.655)	1.35 (0.96-2.93)	0.91 (0.59-1.47)	0.98 (0.61-2.24)
Direct bilirubin	0.73 (0.22-1.96)	0.58 (0.18-1.25)	0.17 (0.09-0.65)	0.21 (0.14-1.1)
Alkaline phosphatase mg/dL, median (IQR)	139 (93-235.5)	127.5 (95-227)	105 (62-161)	79 (61-189)
Creatinine mg/dL, median (IQR)	0.79 (0.7-1)	0.88 (0.65-1.06)	0.74 (0.71-1.23)	0.84 (0.69-0.97)
Triglycerides mg/dL, median (IQR)	174 (63.8-265)	101.25 (81.6-158.9)	179.2 (140-218.4)	204 (96.3-207)
Serum calcium mg/dL, median (IQR)	8.35 (7.8-9.15)	8.2 (7.7-9.1)	8.75 (8.6-8.95)	8.3 (7.55-8.4)

CRP: C-reactive protein; IQR: interqurtilic range; ALT: alanine transaminase; AST: aspartate transaminase.

Finally, a subgroup analysis was performed according to mortality. CRP levels were higher in the population that died (*P*=0.0022), as were total and direct bilirubin levels (*P*=0.05), creatinine (*P*=0.018), and BUN (*P*=0.0007). Similarly, lower PaO2/FiO2 at admission and at 48 hours was associated with death (*P*=0.05 and *P*=0.002, respectively), as was creatinine level at 48 hours (*P*=0.0007). This information is presented in Table 4.

**TABLE 4:** Comparison between variables according to mortality outcomes.

Variable	Mortality (n=14)	No mortality (n=99)	P*
CRP in mg/dL, median (IQR)	25.65 (23.65-30.5)	18.5 (8.6-24.35)	0.0022
Absolute leukocytes (mm3 ), median (IQR)	16500 (11670- 20600)	14200 (10100-17580)	0.2818
Absolute neutrophils (mm3), median (IQR)	15165 (10608-18200)	11700 (7800-14600)	0.1781
Hemoglobin (g/dL), median (IQR)	16 (14.6-17.9)	14.8 (12.9-16.4)	0.1644
Absolute platelets (mm3), median (IQR)	252950 (127400-371000)	269700 (222000-376700)	0.3788
Amylase mg/dL, median (IQR)	1600 (949-407)	1250 (368-2750)	0.3659
Lipase mg/dL, median (IQR)	5083 (1000-9107)	796.5 (295-3282)	0.0719
ALT mg/dL, median (IQR)	226 (59-469)	73.5 (30-275)	0.1215
AST mg/dL, median (IQR)	266.5 (49-416)	70 (30-230)	0.1324
Bilirubin levels in mg/dL, median (IQR)			
Total bilirubin	2.23 (1.13-4.02)	1.27 (0.73-2.89)	0.05
Direct bilirubin	1.19 (0.29-2.34)	0.44 (0.17-1.26)	0.05
Alkaline phosphatase mg/dL, median (IQR)	121 (109-252)	129 (81-190)	0.4422
Creatinine mg/dL, median (IQR)	1.27 (0.68-1.83)	0.81 (0.66-0.99)	0.018
Creatinine mg/dL at 48 hours, median (IQR)	1.36 (1.11-2.08)	0.755 (0.62-0.92)	0.0007
BUN mg/dL, median (IQR)	22.7 (19.5-27.8)	14 (9.6-18.1)	0.0007
Triglycerides mg/dL, median (IQR)	157.4 (74.8-224)	108.95 (80-206.5)	0.8796
Serum calcium mg/dL, median (IQR)	8.25 (7.5-9.2)	8.3 (7.8-8.9)	0.9486
PaO2/FiO2 on admission, median (IQR)	268.5 (223-290)	304 (266-332)	0.05
PaO2/FiO2 at 48 hours, median (IQR)	188.5 (123-248)	267 (228-320)	0.0002
Antibiotic use, n (%)	12 (85.71)	2 (14.29)	0.003

*The *t*-test was used to compare continuous variables with normal distribution, Mann-Whitney test was used to compare continuous variables without normal distribution and χ² test was used to compare proportions.

## DISCUSSION

In our study, we described the clinical characteristics of patients with pancreatic collections secondary to acute pancreatitis in a population managed in a referral hospital in Colombia. The frequency of complications was detailed, showing that 16.4% of patients with acute pancreatitis developed some type of local complication. On the other hand, the most common type of complication was acute necrotic collection, which represented 47.7% of the total collections. Significant differences were found in the laboratory results and treatment according to the type of collection, with more frequent interventional management in more severe complications, as well as a different frequency of mortality according to the type of collection.

Acute necrotic collection was the most common local complication, a finding that contrasts with what has been reported in the literature^12^ ^,^ ^14^ where acute fluid collection is found more frequently. This could be attributed to the type of patients treated at the institution where the study was conducted, which is a referral hospital with available technologies to treat highly complex patients, which could result in a higher influx of patients. A significant proportion of patients with acute necrotic collection underwent interventional management. This finding disagrees with current indications, since intervention in acute collections is preferred when the collection is encapsulated or demarcated, which usually occurs at 4 weeks^15^ ^,^ ^16^. However, this finding may be explained by the presence of infected necrosis, systemic inflammatory response that does not improve despite optimal medical management or failure of conservative management, where early intervention may be indicated, with similar and safe outcomes^17^. Another explanation may be the availability of resources in the institution. CRP, creatinine and BUN levels were higher in patients with acute necrotic collection, which may be related to the persistence of unmodulated systemic inflammatory response, a finding that would also explain the lower PaO2/FiO2 levels in this type of patient. Consistently, these patients had higher scores for respiratory dysfunction and renal dysfunction, which translates into a higher score on the modified Marshall scale and thus higher severity indices. Interestingly, this pattern was not repeated for circulatory dysfunction, perhaps because cardiac injury is associated with pancreatitis particularly in the acute phase^18^. Proposed mechanisms for cardiovascular involvement in acute pancreatitis include metabolic disturbances, direct injury to the pericardium or myocyte membrane, coagulopathy, coronary artery spasm, and hemodynamic instability^19^. Patients with acute necrotic collection usually have a subacute course, and the evidence regarding the relationship between late pancreatitis and cardiovascular disease is inconclusive^18^.

In acute fluid collection the most common therapy was clinical surveillance without intervention, as most tend to resolve spontaneously^15^. Lipase, transaminase, bilirubin and alkaline phosphatase levels were higher in patients with acute fluid collection compared to patients with other types of collections, suggesting that these laboratory findings are not related to the presence of more severe collections. On the other hand, the majority of pseudocysts required interventional management, which contrasts with reported intervention rates of 1.5%^20^. This may be related to the inclusion of patients in the hospital setting, as these patients consult for persistent symptoms, which is the main indication for management of pancreatic pseudocyst. However, the low proportion of patients with pseudocyst included in the study could have altered this result.

Finally, walled-off necrosis received active interventional management in all cases. It is interesting to note that surgical drainage was the preferred method in less than 10% of all collections. This finding is consistent with what has been observed worldwide, where there is a tendency toward stepwise management of pancreatic collections, reserving surgical management for patients in whom minimally invasive therapy has failed^4^ ^,^ ^9^ ^,^ ^21^. Our data suggest that progress is being made in minimally invasive interventional management, which may be advisable due to a lower complication rate and lower morbidity compared to surgical procedures. On the other hand, some studies suggest a higher success rate with endoscopic drainage compared to percutaneous drainage^22^.

Almost half of the collections received antibiotic treatment, although only a minority had microbiological isolation, which can be explained by the difficulty and risk of obtaining a sample for such isolation, as well as the low threshold of medical staff to indicate antibiotic therapy. This is related to the difficulty in differentiating the systemic inflammatory response characteristic of complicated pancreatitis from the deterioration due to active infection, which leads to the early use of empirical antibiotics^23^. Our findings highlight the importance of seeking alternative ways to differentiate between these entities.

The mortality rate was 12.3%, significantly higher than the 1% mortality rate in patients with mild acute pancreatitis^1^ ^,^ ^24^ but lower than the overall reported rate of 15-39% in patients with severe pancreatitis^1^ ^,^ ^4^ ^,^ ^15^ ^,^ ^25^, a subgroup to which our cohort belongs. In the mortality subgroup analysis, CRP, BUN, creatinine levels at admission and at 48 hours, PaO2/FiO2 at admission and at 48 hours were statistically significantly associated with mortality, suggesting that changes in these laboratories indicate strict follow-up and consideration of early interventional management. A possible explanation is the association of these findings with the persistence of systemic inflammatory response and multiorgan dysfunction, which are the major causes of mortality in patients with severe acute pancreatitis^15^ ^,^ ^26^. Most deaths from this causes occur in the first 2 weeks, while the remaining late deaths are due to complications secondary to infection of pancreatic necrosis and complications related to interventions^15^ ^,^ ^24^. The higher use of antibiotics in the population that died may be interpreted as an indicator of superinfection or inadequate evolution. Nevertheless, the levels of leukocytes and neutrophils, a possible surrogate of infection, were not statistically significantly associated with mortality, which may be explained by their elevation in patients with systemic inflammatory response and not necessarily by the presence of active infection.

Our study included all patients with acute pancreatic collections in a given time period and, to our knowledge, is the largest cohort of this population in Latin America. However, there are limitations that should be acknowledged. Our results represent the experience of a single hospital with a high level of complexity, limiting the external validity in other institutions with a lower level of complexity. Further studies are needed to assess whether the results are similar in other institutions in Latin America. The data was collected retrospectively from a database of electronic medical records and there may be missing data for some patients. However, data were complete for the vast majority of patients, and the rate of missing data was less than 5%. Also, the data was collected in a standardized format.

## CONCLUSION

Pancreatic collections secondary to acute pancreatitis are a relatively common complication of pancreatitis. Most fluid collections do not require intervention, in contrast to acute necrotic collections and walled-off necrosis. Surgical management is now less frequent compared to minimally invasive and combined management, as reported in other parts of the world. Antibiotic use is high despite low microbiological isolation. CRP levels, BUN, renal dysfunction, respiratory dysfunction and antibiotic use were significantly associated with mortality.
